# Pembrolizumab-related systemic myositis involving ocular and hindneck muscles resembling myasthenic gravis: a case report

**DOI:** 10.1186/s12883-019-1416-1

**Published:** 2019-08-05

**Authors:** Hikaru Kamo, Taku Hatano, Kazuaki Kanai, Nozomi Aoki, Daiki Kamiyama, Kazumasa Yokoyama, Masashi Takanashi, Yuri Yamashita, Yasushi Shimo, Nobutaka Hattori

**Affiliations:** 1 Department of Neurology, Juntendo University Graduate School of Medicine, 2-1-1 Hongo, Bunkyo-ku, Tokyo, 113-8421 Japan; 2 Department of Neurology School of Medicine, Fukushima Medical University, Fukushima City, 960-1295 Japan; 3 Department of Research and Therapeutics for Movement Disorders, Juntendo University Graduate School of Medicine, Tokyo, Japan

**Keywords:** Pembrolizumab, Necrotizing myositis, ICPIs-related systemic myositis, Ocular myositis, Immune-related adverse events, Myasthenia gravis

## Abstract

**Background:**

Pembrolizumab is an immune-checkpoints inhibitor that enhances the immune response against cancer cells and therefore is useful for the treatment of several carcinomas. However, pembrolizumab sometimes perturbs the immune system resulting in various autoimmune neurological complications. In this situation, autoimmune myositis due to pembrolizumab is a rare but not-negligible complication. Here, we report two cases of autoimmune myositis due to pembrolizumab, with systemic myositis involving levator palpebrae superioris, extraocular and hindneck muscles.

**Case presentation:**

Case 1 was a 78-year-old man with advanced urinary cancer referred to the neurological ward presenting with bilateral ptosis, restriction of eye movements, dropped head and weakness in the lower extremities after pembrolizumab administration. His blood examination showed elevated serum levels of creatine kinase with positive anti-PM-Scl 75 and anti-signal recognition particle antibodies. Needle electromyography and MRI suggested systemic inflammatory myopathy. There were no findings to indicate myocardial involvement on electrocardiogram or echocardiogram. Administration of intravenous methylprednisolone following plasma exchange ameliorated creatine kinase levels and inhibited the progression of clinical symptoms. Case 2 was a 72-year-old female with lung cancer and multiple metastasis, including lymph nodes and brain. She presented with back pain, right-sided ptosis, weakness of her neck extensors and flexors and elevated serum creatine kinase after receiving pembrolizumab. Although myositis specific autoantibodies were negative, needle electromyography and MRI suggested systemic inflammatory myopathy and muscle biopsy indicated necrotizing myopathy. There were no signs indicating heart dysfunction and her electrocardiogram was normal. Clinical symptoms and serum creatine kinase levels were ameliorated after the administration of intravenous methylprednisolone.

**Conclusions:**

Both cases showed atypical extensive inflammatory myositis including levator palpebrae superioris, extraocular and hindneck muscles, resembling myasthenia gravis (MG), but they did not have MG-related antibodies. Edrophonium test was negative and showed no daily fluctuation. Two previously reported cases also presented with systemic necrotizing systemic myositis involving extraocular and facial muscles caused by pembrolizumab. Idiopathic inflammatory myositis evolving levator palpebrae superioris and ocular muscles is quite rare; however, myositis due to immune-checkpoint inhibitors may preferentially involve these muscles. This case report will alert physicians to the possibility of systemic inflammatory myopathy evolving levator palpebrae superioris, extraocular and hindneck muscles mimicking MG due to pembrolizumab.

## Background

Pembrolizumab is classified as an immune checkpoint molecule inhibitor (ICPI) and is an IgG 4 monoclonal antibody that recognizes the programmed cell death 1 (PD-1) receptor, enhancing pre-existing immune responses [1]. Upregulation of the immune system by pembrolizumab was reported to be associated with various autoimmune neurological complications, such as myasthenic gravis (MG), myositis and polyneuropathy [2]. In this situation, autoimmune myositis due to pembrolizumab is a rare but not-negligible complication. The specific findings of autoimmune myositis due to pembrolizumab are necrotizing myositis with elevated serum creatine kinase (CK) levels. Some patients with necrotizing myositis associated with pembrolizumab have involvement of the systemic muscles including, levator palpebrae superioris, extraocular, hindneck, proximal upper and lower limb muscles, resembling MG, whereas pembrolizumab-induced MG is sometimes complicated with myositis [2]. Thus, it is important to distinguish between necrotizing myositis and MG due to pembrolizumab. Here, we report two cases of pembrolizumab-induced systemic myositis involving levator palpebrae superioris, extraocular and hindneck muscles, resembling MG. However, they did not have any abnormalities of the neuromuscular junction or MG-related antibodies.

## Case presentation

### Case 1

**Fig. 1:**
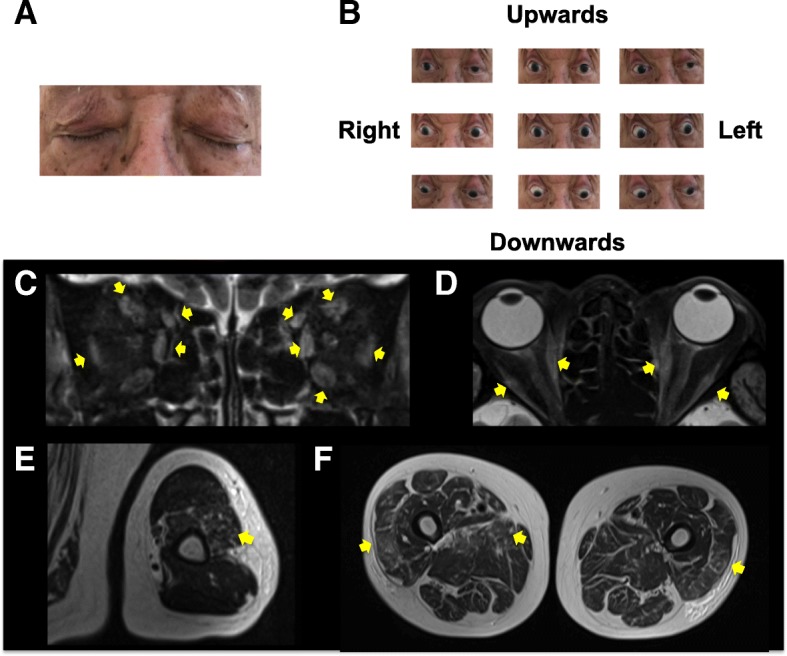
Ptosis, eye movements and muscle MRI in case 1. On admission, case 1 exhibited bilateral ptosis (**a**), and restricted eye movements, particularly for upward gaze (**b**). Hyperintense signal and enhancement on short T2 inversion recovery (STIR) in bilateral orbicularis oculi muscle: coronal view (**c**) and axial view (**d**), brachial muscle (**e**) and femoral muscle (**f**). Yellow arrows indicate inflammation changes in muscles

A 78-year-old man with urinary cancer was consulted to neurological service and referred to the neurological ward presenting with generalized weakness. He was diagnosed with renal, pelvis, and ureter cancer with multiple metastasis of lymph nodes 2 years before consultation. The patient’s tumor worsened, despite three courses of methotrexate, vinblastine, adriamycin and cisplatin, with three courses of gemcitabine and cisplatin. After chemotherapy, pembrolizumab was administered for the enlargement of lymph nodes. Serum levels of CK were increased to 817 U/L (normal range 57–240) at the second course of pembrolizumab, and, in the following several weeks, he presented with bilateral ptosis (Fig. 1a), restriction of eye movements (Fig. 1b), dropped head and lower extremities, without daily fluctuation. Deep tendon reflexes were diminished with left lateral femoral weakness. His blood examination showed elevated serum levels of CK 7765 U/L and aldolase 91.8 U/L (normal range < 6.0 U/L). Anti-PM-Scl 75 and anti-signal recognition particle (SRP) antibodies were positive, but no other myositis specific autoantibodies, including Jo-1, ARS, MDA5, TiF1γ, Mi-2, Ku, PL-7, PL-10, OJ, EJ, or MG-related antibodies, including AChR and muscle specific kinase (MuSK) were observed. Repetitive nerve stimulation test (RST) revealed no significant decrement in any muscles tested. Needle electromyography revealed low amplitude polyphasic motor unit potentials with early recruitment in right biceps brachii and vastus lateralis. Fibrillation potentials, increased insertional irritability and repetitive discharges were also found, suggesting inflammatory myopathy. Hyperintense signal and enhancement on short T2 inversion recovery (STIR) was observed for both sides of the orbicularis oculi, brachial and femoral muscles (Fig. 1c–f). The patient presented with omnidirectional eye movement restriction and MRI revealed inflammatory changes in each orbital oculi muscle so that gaze restrictions were inferred to be related to ocular myositis. Evidence of inflammation in limbs by MRI indicated the weakness in neck extensor and lower extremities resulting in a manual muscle testing (MMT) score of 4 were caused by myositis. There were no signs indicating heart dysfunction, such as heart failure and arrhythmia. Edorophonium test was negative. Pembrolizumab-related myositis was suspected, and administration of intravenous methylprednisolone (IVMP) following plasma exchange (PE) ameliorated CK levels to 984 U/L and inhibited the progression of clinical symptoms. However, the patient died due to a worsening metastasis of cancer. The patient’s family declined an autopsy.

### Case 2

**Fig. 2:**
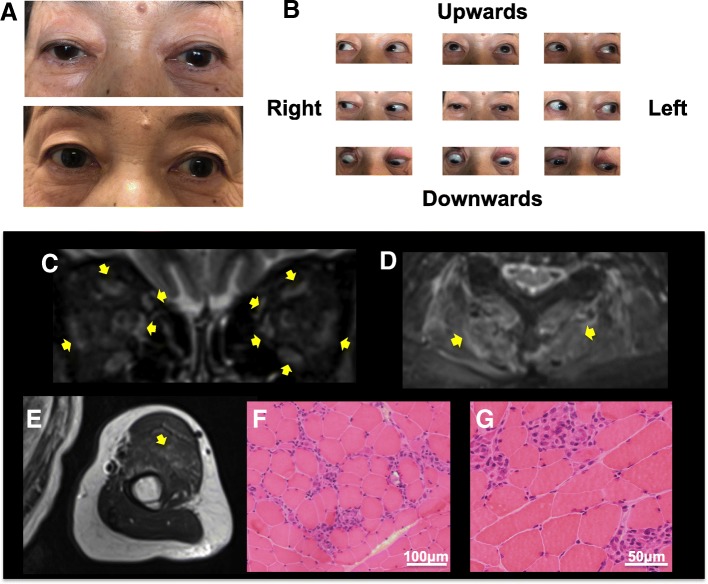
Ptosis, eye movements, muscle MRI and pathological findings in case 2. On admission, case 2 exhibited bilateral ptosis (Above). After treatment, ptosis was improved (Below) (**a**). Restriction of eye movements on admission (**b**). Hyperintense signal and enhancement on short T2 inversion recovery (STIR) in bilateral orbicularis oculi muscle: coronal view (**c**) posterior cervical muscle (**d**), and brachial muscle (**e**). Yellow arrow indicates inflammation change. Muscle biopsy shows muscle fiber necrosis and regeneration with sparse inflammatory infiltrate with hematoxylin and eosin staining. (**f**, **g**)

A-72-year old female with lung cancer was consulted to neurological service presenting with back pain and elevated serum CK. She was diagnosed with lung cancer with multiple metastasis of the lymph nodes and brain, 6 months before consultation. After stereotactic radiotherapy for brain metastasis, she received pembrolizumab for the enlargement of a lymph node. Serum levels of CK increased to 1817 U/L after the second course of pembrolizumab and she presented with back pain, right-sided ptosis, restricted eye movements (Fig. 2a, b) and weakness of her neck extensors and flexors. Her blood examination showed elevated serum levels of CK 1844 U/L and aldolase was elevated to 18.2 U/L. Myositis specific autoantibodies including Jo-1, ARS, MDA5, TiF1γ, Mi-2, Ku, PL-7, PL-10, OJ, EJ, HMGCR, SRP and myasthenic gravis related antibodies including anti AChR and MuSK antibody were negative. She had no signs indicating heart dysfunction and electrocardiogram revealed no specific abnormal findings. EMG revealed myogenic change with acute denervation in the right biceps brachii and trapezius without waning in the orbicularis oculi muscle. Hyperintense signal and enhancement on STIR was observed in both sides of the orbicularis oculi muscle, posterior cervical muscles, brachial and femoral muscle (Fig. 2c, d, e). Although her symptoms were insignificant, MRI showed diffuse inflammatory changes. Muscle biopsy showed muscle fiber necrosis and regeneration with sparse inflammatory infiltration, indicating she had immune-mediated necrotizing myopathy (Fig. 2f, g). Edorophonium test was negative and there was not daily fluctuation. Thus, she was diagnosed with myositis due to pembrolizumab. Her clinical symptoms including ptosis, eye movement restriction and serum CK levels were ameliorated (Fig. 2a) soon after the administration of IVMP. She was discharged and received oral PSL 0.5 mg/kg/day and tapering thereafter.

## Discussion and conclusions

Immune check points are molecules that participate in the regulation and maintenance of immunologic homeostasis. Inhibitors of immune check points, including PD-1 and cytotoxic lymphocyte-associated protein 4 (CTLA-4), trigger the T-cell-mediated attack of cancer cells and are used for the treatment of several tumors. However, these drugs are rarely associated with the induction of various immune-related adverse events (irAE). Overall neurological complications were observed in 6% of irAE but myositis occurred in less than 1% of cases [2]. Myositis due to irAE is thought to be a pathomechanism whereby activated CD8+ T lymphocytes directly attack their own muscle. In case 1, anti-PM-Scl 75 and anti-SRP antibodies were positive. Anti-PM-Scl 75 antibody is usually detected in sera from patients with polymyositis (PM), systemic sclerosis (SSc) and PM/SSc overlap syndromes [3]. Anti-SRP antibody is usually associated with typical polymyositis or necrotizing myositis, which presents as inflammation limited to the proximal limbs and trunk. Although we could not assess the muscle histology of case 1, he might have had necrotizing myositis because of anti-SRP antibody positivity.

Our two cases presented with atypical extensive inflammatory myositis including the hind neck, levator palpebrae superioris and ocular muscle, resembling MG. MG can be triggered by ICPIs, and a case with myositis overlapping with MG was reported [2]. However, our cases did not respond to edrophonium test, were negative for MG-related antibodies and RST did not reveal significant decrement, suggesting neuromuscular junctions were not affected. Case 2 and two previously reported cases (Table 1) also presented with systemic necrotizing myositis involving extraocular and hind neck muscles [4, 5]. Moreover, some patients treated with ipilimumab, a CTLA-4 inhibitor, exhibited ocular myositis with or without systemic myositis [6]. Touat et al. reported ten cases of irAE related myositis who lacked autoimmune-myositis related antibodies although 70% of patients presented with ocular myositis. Although 40% of patients had confirmed or possible myocarditis [7], we did not find any evidence of myocardial involvement in both of our cases.

**Table 1:** Pembrolizumab induces systemic myositis presenting as ocular myositis

	Our case 1	Our case 2	Case1 [4]	Case2 [5]
Age	78	72	78	86
Sex	M	F	M	F
Carcinoma	pelvis and ureter cancer	lung cancer	metastatic melanoma	metastatic melanoma
Myositis symptom onset	7 days after second injection	2 weeks after second injection	2 weeks after second injection	4 days after second injection
Initial symptom	ptosis	back pain, right-sided ptosis and weakness of neck	bulbar weakness	fatigue, left ptosis and ophthalmoplegia
CK (IU/L) at the 1st visit	6416	1603	1284	1499
EMG finding	myogenic change	myogenic change	myogenic change	myogenic change
Serum antibody	PM-Scl 75, SRP	negative	negative	negative
Anti-AChR antibody	negative	negative	negative	negative
MG overlap	–	–	–	–
Pathology	–	necrotizing myositis	necrotizing myositis	necrotizing myositis
Initial treatment	IVMP	IVMP	PSL 1 mg/kg/day	IVMP
Second treatment	PLEX	Oral PSL	PLEX	PLEX
Outcome	dead due to cancer	complete clinical recovery	dead due to respiratory weakness	complete clinical recovery

*AChR* acetylcholine receptor, *CK* creatine kinase, *IVMP* intravenous methylprednisolone, *N.A.* not available, *PLEX* plasma exchange, *PSL* prednisolone, *SRP* signal recognition particle

Myositis in our cases responded favorably to IVMP with or without plasma exchange. Vallet et al. reported a patient with ICPIs-related systemic myositis (ICPIrsm) ameliorated by IVMP with plasma exchange [5], whereas Haddox et al. reported a case with refractory ICPIrsm [4]. Responses to immunotherapy might differ between patients; therefore, physicians should carefully observe the clinical course of patients with ICPIrsm, even if intensive treatment was performed.

Myositis due to pembrolizumab is not usually associated with paraneoplastic antibodies as seen in our case 2 [4, 5, 7]. Interestingly, case 1 had several autoimmune myositis-related antibodies. Thus, myositis in case 1 might have been caused by idiopathic inflammatory myositis related to anti-PM-Scl 75 and anti-SRP antibodies. However, idiopathic inflammatory myositis involving ocular muscles is rare [8]. Whereas myositis due to ICPIs may preferentially involve these muscles. Considering the symptoms and clinical course of case 1, we believe that his myositis was associated with immunoperturbation due to pembrolizumab.

In conclusion, physicians and neurologists should be aware that ICPIrsm can involve the levator palpebrae superioris, extraocular and hindneck muscles, mimicking MG.

## Data Availability

All the relevant raw data in the current study will be freely available to any scientist wishing to use them without breaching participant confidentiality for non-commercial purposes.

## Abbreviations

AChR: Acetylcholine receptor
CK: Creatine kinase
ICPIrsm: ICPIs-related systemic myositis
ICPIs: Immune checkpoint molecule inhibitors
irAE: Immune-related adverse events
IVMP: Intravenous methylprednisolone
MHC-II: Major histocompatibility complex class II
MMT: Manual muscle testing
MuSK: Muscle specific kinase
PD-1: Programmed cell death 1
PE: Plasma exchange
RST: Repetitive nerve stimulation test
SRP: Signal recognition particle
STIR: Short TI inversion recovery
TCR: Receptor of T lymphocytes
